# Medication supply chain management through implementation of a hospital pharmacy computerized inventory program in Haiti

**DOI:** 10.3402/gha.v8.26546

**Published:** 2015-01-22

**Authors:** Michelle R. Holm, Maria I. Rudis, John W. Wilson

**Affiliations:** 1 Department of Pharmacy, Mayo Clinic, Rochester, MN, USA; 2 Department of Emergency Medicine, Mayo Clinic, Rochester, MN, USA; 3 Division of Infectious Diseases, Department of Internal Medicine, Mayo Clinic, Rochester, MN, USA

**Keywords:** computer program, pharmacy, inventory, medication management system, global health inventory system solution

## Abstract

**Background:**

In the aftermath of the 2010 earthquake in Haiti, St. Luke Hospital was built to help manage the mass casualties and subsequent cholera epidemic. A major problem faced by the hospital system was the lack of an available and sustainable supply of medications. Long-term viability of the hospital system depended largely on developing an uninterrupted medication supply chain.

**Objective:**

We hypothesized that the implementation of a new Pharmacy Computerized Inventory Program (PCIP) would optimize medication availability and decrease medication shortages.

**Design:**

We conducted the research by examining how medications were being utilized and distributed before and after the implementation of PCIP. We measured the number of documented medication transactions in both Phase 1 and Phase 2 as well as user logins to determine if a computerized inventory system would be beneficial in providing a sustainable, long-term solution to their medication management needs.

**Results:**

The PCIP incorporated drug ordering, filling the drug requests, distribution, and dispensing of the medications in multiple settings; inventory of currently shelved medications; and graphic reporting of ‘real-time’ medication usage. During the PCIP initiation and establishment periods, the number of medication transactions increased from 219.6 to 359.5 (*p*=0.055), respectively, and the mean logins per day increased from 24.3 to 31.5, *p*<0.0001, respectively. The PCIP allows the hospital staff to identify and order medications with a critically low supply as well as track usage for future medication needs. The pharmacy and nursing staff found the PCIP to be efficient and a significant improvement in their medication utilization.

**Conclusions:**

An efficient, customizable, and cost-sensitive PCIP can improve drug inventory management in a simplified and sustainable manner within a resource-constrained hospital.

The World Health Organization estimates that a third of the population in developing countries does not have access to essential medicines (1). In addition, many governments and health care facilities do not have a sustainable, uninterrupted, drug supply chain process. Unreliable monitoring of medication utilization, inconsistent supplies of medications, concerns for medication integrity, and illicit drug diversion among hospital staff for personal use (2, 3) all highlight the many supply chain problems in under-resourced settings.

In the aftermath of the January 2010 Haiti earthquake, St. Luke Foundation/Nuestros Pequeños Hermanos built St. Luke Hospital, an adult field hospital, to help manage the overabundance of new adult patients with trauma injuries, cholera infection, and other acute illnesses. This hospital was erected adjacent to the already established St. Damien’s Children’s Hospital, Haiti’s only referral pediatric hospital. A major problem faced by the Haitian-led organization was the lack of an available and sustainable supply of medicines (and other medical supplies). Drug costs had continued to rise (4, 5), and approximately half of hospital budgets were used primarily for acquisition of medications (2, 3). Long-term survival and sustainability of the health care facilities themselves depended in large part on developing an uninterrupted medication supply chain. Development of a simple inventory and supply chain management system was needed at St. Luke Hospital to help keep the critical drugs on the shelves and prevent low-usage medications from expiring.

The goal of our collaborative project was to develop a simple and sustainable electronic pharmacy supply chain management system at St. Luke Hospital to track drug acquisition, storage, distribution, and utilization. We describe the development and implementation, staff education, and outcomes achieved with the Pharmacy Computerized Inventory Program (PCIP).

## Methods

### Setting/time/design

St. Luke Hospital is located in Tabarre, which is within Port-Au-Prince. It consists of an adult hospital and an outpatient clinic with an annual census of 150,000 patient visits per year. The emergency department has approximately 150 visits per day; the ICU has eight beds; and the cholera center has treated 20,000 patients since its inception in 2010. St. Luke Hospital is directly affiliated with the St. Damien’s Children’s Hospital.

As with many medical centers operating within resource-constrained communities around the world, St. Luke Hospital is plagued by personnel and pharmaceutical supply shortages. With such shortcomings we hypothesized that a simple and sustainable electronic pharmacy supply chain management system, complete with an off-line alternative, could be developed and implemented to allow a more effective and sustainable hospital-based pharmacy practice and supply chain management system.

We conducted our research by examining how medications were being utilized and distributed before and after the implementation of PCIP. Prior to the PCIP implementation medication transactions were only documented on occasion and records were not organized in a manner conducive for retrieval. Personnel were not assigned to manage the medication inventory due to lack of a formalized process, therefore medication ordering was completely based on inference and presumption of which medications were currently being utilized and which medications may be needed in the future.

Given the internet capacity at St. Luke’s and the limited pharmacy personnel, an electronic system was identified as a possible solution for inventory and supply chain management concerns. Frequent power and internet outages experienced throughout Haiti verified a backup card-based system would be required if, in fact, PCIP was deemed successful. PCIP was composed in both Haitian Creole and English due to the preponderance of Creole and French languages to accommodate both the Haitian hospital pharmacists and foreign volunteers who frequently volunteered at St. Luke’s.

We measured the number of documented medication transactions in both Phase 1 and Phase 2 along with measuring user login data to determine if a computerized inventory system would be beneficial in providing a sustainable, long-term solution to institutional medication management needs.

### Process

Our project was conducted in four phases over a period of 1 year (March 2011 to February 2012). Phase 1 consisted of performing a needs assessment of supply chain management at St. Luke Hospital. Phase 2 comprised the development and testing of a computerized software system to fulfill supply chain needs. During Phase 3, we implemented PCIP and educated hospital staff on its use. Finally, in Phase 4, we measured the utilization of the PCIP system at the initiation of the program and 6 months after its implementation (August 10, 2011, to February 10, 2012) to quantify the number of transactions processed in the PCIP system. Our institutional review board reviewed in accordance with the US Code of Federal Regulations, 45 CFR 46.

### Phase 1 – needs assessment

During our initial visits to St. Luke Hospital, the medical director identified that a pharmacy supply chain management system was a top strategic institutional priority in order to maintain a viable operation at St. Luke Hospital, and to help with sustainability of growth of health care systems within the St Luke Medical Missions. Our pharmacists spent time with the medical director, pharmacy staff, nursing, and inventory staff to complete a needs assessment. The assessment included value stream mapping of existing processes for medication supply chain management, identifying obstacles surrounding drug procurement and distribution within the facility, and identifying characteristics of a sustainable drug supply chain management system.

### Phase 2 – development of PCIP

After formulating the requirements of a supply chain management system, we looked for an inexpensive data management system that met identified needs and would be able to be implemented in short order. We utilized an inventory management system by PlanetJ Corporation, maker of Web Object Wizard. Over a period of 1–2 months, we developed the hospital drug inventory system according to the desired characteristics, and completed database programming and testing. The database was populated with all of the available medications at St. Luke Hospital.

The construct of the PCIP was to be an electronic medication request platform from a listing of locally available hospital drugs; a decision process for drug approval or denial, based predominately on existing drug availability; an electronic real-time debit of the inventory; and the filling of drug requests at the point of care. To allow for situational awareness of the status of requested medications, and to encourage consistent use of the PCIP, information was to be visible to all hospital users on computers in patient care areas. Front end users were to be nurses in patient care areas, and back end users were to be designated pharmacy personnel who would fill requests at regular intervals throughout the day, based on the availability of the drugs in stock. The program was developed in both Haitian Creole and English for use by both the Haitian staff and by visiting foreign aid workers.

As we worked through the end user experience, we examined the efficiency of the user interface to minimize steps/screens to request and fill the desired medication. We conducted pilot testing both on-site and remotely (Haiti and US), and made any needed changes to the system before full implementation was initiated.

Because there is a high frequency of power outages in Haiti and variability in internet speed, we also developed a ‘paper’ backup system to support the PCIP. This system consisted of two distinct standardized laminated stock cards with medication name, strength, and formulation. The first was a ‘low stock inventory’ and the second was an ‘out of stock/therapeutic alternatives’ card. The low stock inventory card was used by nurses to request a medication by placing it in the pharmacy bin for pickup. When the pharmacy received the request, the medication would be filled if it was in stock. If however, the pharmacy was out of stock of that particular drug, the pharmacist would reference the out of stock card to identify therapeutic alternatives for that agent listed on its reverse side, and dispense the alternative. This out of stock resource card was also able to be utilized in concert with the PCIP.

### Phase 3 – implementation of PCIP and user education

After remote and on-site pilot testing, the PCIP program was launched on designated servers at St. Luke Hospital, and was accessible to hospital staff on the internet. During the implementation period, we utilized rapidly cycled plan-do-study-act (PDSA) testing, a quality improvement methodology for action-oriented learning and systems improvements (6).

On-site education about PCIP used principles of adult active learning, and consisted of small group sessions of Haitian pharmacy, nursing, administrative personnel, and physician staff, led by our Mayo Clinic team. Learners demonstrated their practical understanding of the PCIP system through return demonstrations during the small group training sessions. Continued training/education ensued during the next several visits to Haiti over a period of approximately 6 months (at 1 month intervals) to ensure continued proficiency, and to educate new staff.

### Phase 4 – outcomes and data analysis

To objectively quantify the utilization of the new inventory and supply chain management system and to determine its sustainability, we measured the pharmacy and physician staff utilization of PCIP as well as the number of transactions processed in PCIP at the initiation of the program (August 10, 2011) and 6 months after its implementation (February 10, 2012). Descriptive statistics were performed for categorical data and were summarized as frequencies and means, as applicable. Differences in outcome parameters between the two time periods are expressed as proportions, with a two-sided *p<*0.05 being significant.

## Results

### Phase 1 – needs assessment

Results of the needs assessment for a supply chain management system included requirements for tracking of expenses on pharmaceutical agents; a mechanism to improve the efficiency of ordering medications for the entire hospital; and storage of a variety of medication data. It also needed to be accurate and reflect real-time inventory, as well as functional for its users in terms of simplicity and functionality in multiple languages, including Haitian Creole (Table 1). These were elements deemed to be necessary for a sustainable system.

**Table 1:** Needs assessment: key functionality requirements of a computerized supply chain management system

Tracking of expenditures	Cost-analysis on elements of medication use processes. Graph and forecast future medication needs based on historical usage.
Efficiency and scaling	Order and track supplies in all hospital settings (i.e. emergency department, cholera center, intensive care unit, inpatient).
Comprehensive medication data	Organize by name of drug, route of administration, country the medication ships from, therapeutic category, critical drugs, narcotics, and medications with low stock inventory.
Accuracy and availability	Real-time inventory.
Cultural and literacy contextualization	Design that could easily be adapted to the user’s educational level. Simple and easy to learn (visual). Simple to use. Multiple language capability.

A needs assessment was completed in Phase 1 to verify both the medical director and our institution adequately addressed all of St. Luke needs before developing a viable solution to the medication supply chain management issues.

### Phase 2 – development of PCIP

**Fig. 1:**
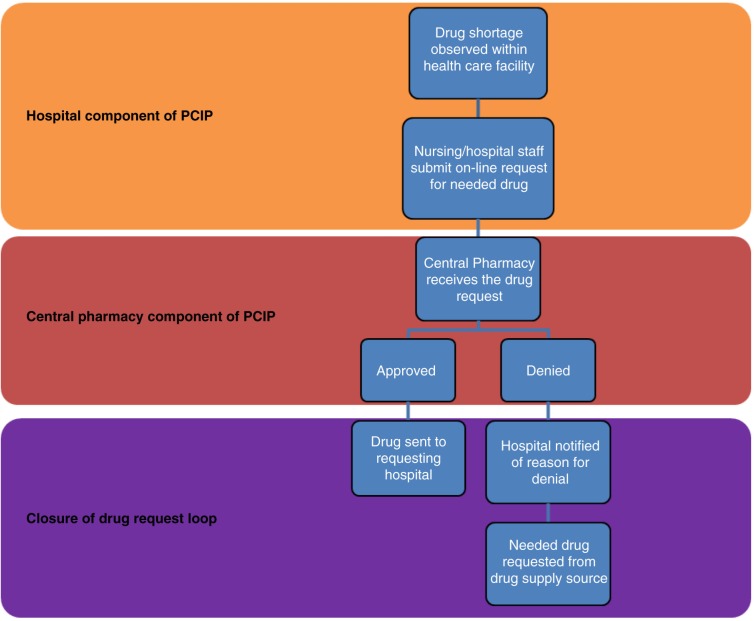
Process flow for medication requests in PCIP.

**Fig. 2:**
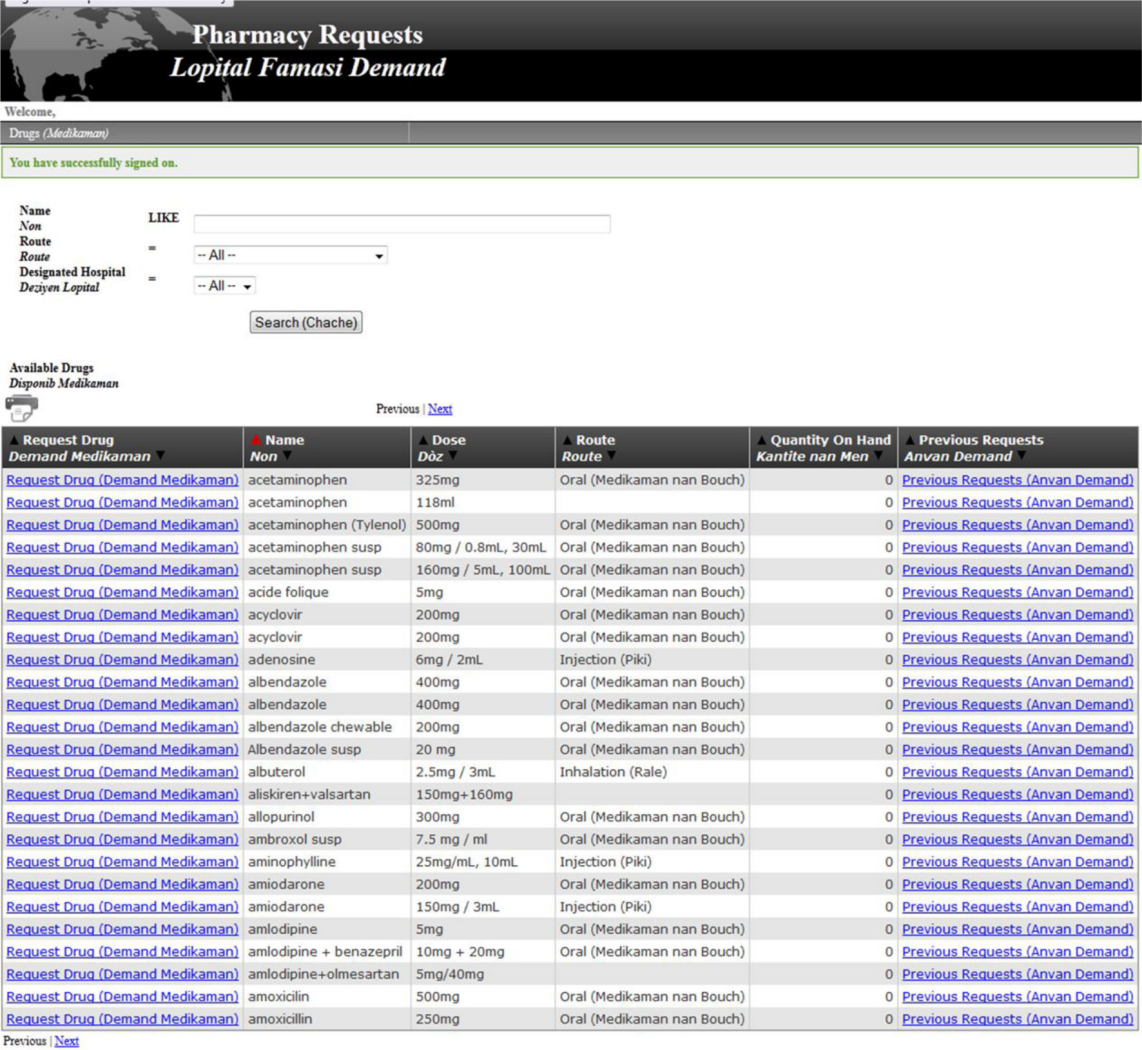
Pharmacy computerized information system: nursing online requests to pharmacy. 1) The nursing staff can search for the drug by typing in the first few letters or by scrolling through the list of medications in alphabetical order. 2) After locating the drug needed the nurse can see the last time it was requested, how much the pharmacy currently has on hand, and can place the request for the desired amount, along with a comment field for certain requests (such as: needed by tomorrow). 3) The pharmacy receives the request electronically and verifies the product on the shelf matches the request. 4) The pharmacy staff then approves or denies the request which the nurse can visualize when he/she goes back into the ‘completed requests’ tab along with a comment field option (e.g. manufacturer shortage, unable to fill as requested).

**Fig. 3:**
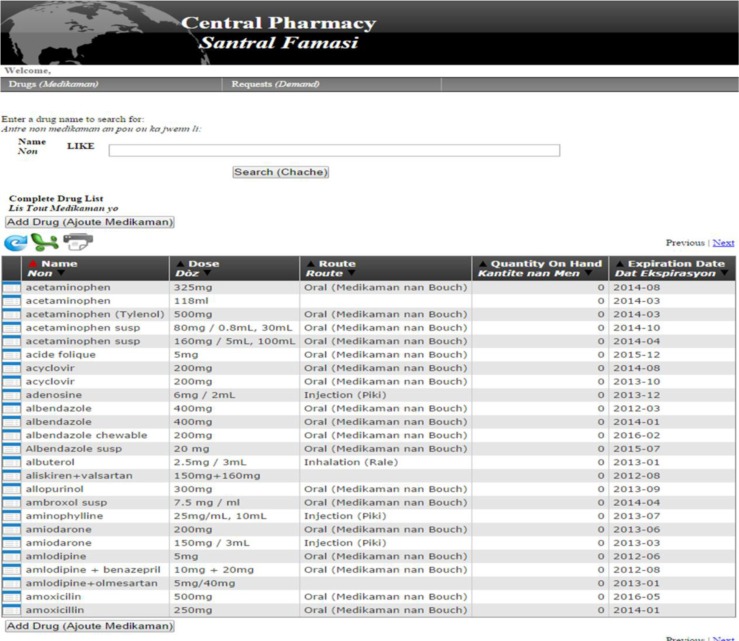
Pharmacy computerized information system: central pharmacy medication inventory. Adding a new drug by the pharmacy staff when a shipment arrives involves searching for the drug by the first few letters or scrolling (similar to the nursing staff side) and then selecting the desired drug. After selecting the drug of choice, the pharmacy staff may change the quantity, expiration date, etc. If the drug is a new drug, then each field is filled in by the pharmacy staff.

**Fig. 4:**
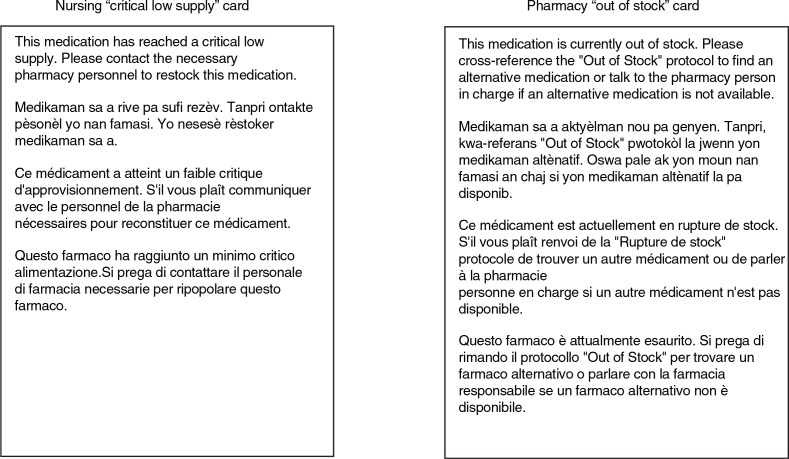
Sample stock cards for nursing and pharmacy staff for non-web based use of the inventory system. If/when the internet fails a set of stock cards allows the hospital system to continue running smoothly by providing the means for nursing staff to request a medication and the pharmacy to fill the medication or quickly find an alternative medication (listed on the back) before sending the requested medication (or alternative) back to the designated hospital.

The PCIP was developed based on the completed needs assessment. The medication request and fill process is illustrated in Fig. 1. Figure 2–4 illustrate screenshots as viewed by individuals using the PCIP system during the medication request and fill process. Figure 2 shows the screens for nurses and providers to enter online medication requests. Figure 3 depicts the screens used by central pharmacy to add a new medication to the inventory, and to maintain or adjust quantities of medications available. Figure 4 displays the off-line medication stock request cards designed for use in circumstances when the web-based PCIP program is unavailable.

### Phase 3 – implementation of PCIP and user education

Once the PCIP system was developed, we educated approximately 75 end users of the various processes in PCIP. End users consisted of nurses, as well as pharmacy staff. Teaching the system to the Haitian pharmacy and nursing staff took approximately 10–15 min per person. Education was completed in small group and individual sessions, with assistance of a translator. During the implementation/education phase, we optimized PCIP screens using PDSA cycles.

### Phase 4 – outcomes and data analysis

**Fig. 5:**
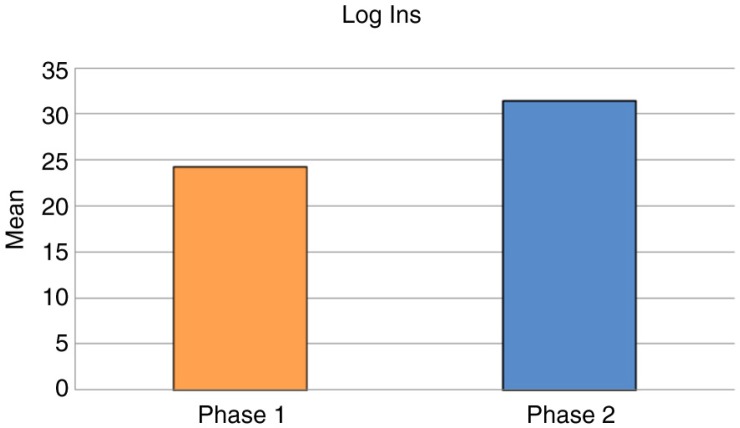
Comparison of logins in pharmacy computerized information system during initiation and establishment periods. The mean (SE) logins per day for Phase 1 and Phase 2 were 24.3 (0.8) and 31.5 (0.7), *p*<0.0001, respectively. Phase 1 (initiation period) consisted of a 6-month period from August 10, 2011, to February 10, 2012. Phase 2 (establishment period) consisted of a 6-month period from February 10, 2012, to August 10, 2012.

**Fig. 6:**
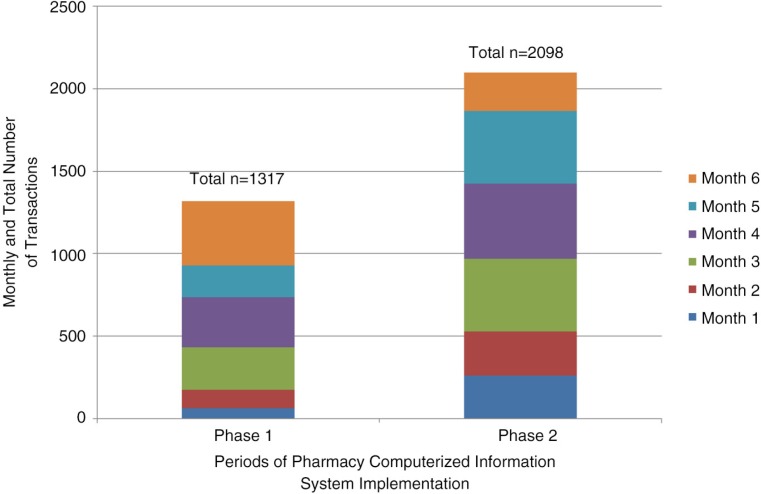
Comparison of transactions in pharmacy computerized information system during Phase 1 (initiation period) and Phase 2 (establishment period). For transactions, the mean (SE) transactions per month for the Phase 1 and Phase 2 periods were 219.6 (42.9) and 359.5 (42.9), *p*=0.055, respectively. Phase 1 consisted of a 6-month period from August 10, 2011, to February 10, 2012. Phase 2 consisted of a 6-month period from February 10, 2012, to August 10, 2012.

In total 4,139 transactions were recorded over the course of Phase 1 and Phase 2. Staff utilization of the PCIP significantly increased over time as reflected by an increase in the mean (SE) number of logins into the system to request a medication in Period 2 compared to Period 1 [31.5 (0.7) vs. 24.3 (0.8), *p*<0·0001] (Fig. 5). The number of transactions processed in PCIP at the initiation of the program and 6 months after its implementation also increased in Period 2 [359.5 (42.9) vs. 219.6 (42.9), *p*=0·055] (Fig. 6). Total drugs in the inventory system to date is reported as 407 distinct drug records compared to zero at the start of program initiation.

## Discussion

Our study demonstrated the feasibility of developing and implementing a sustainable web-based PCIP for improved supply chain management of medications in a large hospital in an under-resourced country such as Haiti. Initial successful adoption and the subsequent significant increase in both PCIP access and overall medication transactions over a period of 6 months suggest that the program was easy enough to learn and use. The PCIP requires minimal ‘mouse clicks’ and provides information about drug usage, drug shortages, expiring drugs, and inventory status. Although we do not report data on cost savings, continued use of the system and anecdotal reports by the hospital administrative staff suggest that PCIP continues to be an effective tool to manage inventory more efficiently at St. Luke Hospital, and as such, was adequately tailored to meet the stated institutional need for such a tool. Hospital administration has the ability to make major changes behind the scenes when needed which keeps the screen shots simple and straightforward for the average daily user. Quick access to data generated within PCIP allowed greater insight into medication utilization patterns and allowed pharmacy and administrative staff to forecast inventory needs, resulting in fewer stock interruptions.

We observed additional benefits of the system that were not entirely unexpected. Although the number of medications the pharmacy stocked increased considerably after PCIP implementation, hospital staff spent less time on medication inventory and supply issues. Nurses spent more dedicated time on direct patient care when medications were reliably in stock at their point of care. Staff satisfaction also seemed to increase as each person’s role became more manageable. Physicians were able to better care for their patients by knowing which medications were available. Pharmacy staff were better able to manage formulary shortages knowing which alternative medications were available for use in its place. Professional collaborative relationships between physicians and nurses improved as a function of greater nursing presence at the bedside. Improved awareness and tracking of inventory levels also reduced drug diversion as the PCIP was essentially a monitoring tool used to track each medication throughout the usage process along with personnel who monitored the medication inventory each day.

Unfortunately data regarding drug diversion was not quantified before and after the system was put in place. Perception of institutional leadership was that there was a reduction in medication diversion by staff after PCIP implementation. Future research is needed, however, to determine if a specific cause and effect relationship exists between the implementation of a PCIP and the decrease of drug diversion.

Despite the successes of the PCIP implementation, we did encounter a number of barriers. Access to electricity and the internet can be sporadic and unreliable. When this occurred in the hospital, it interrupted use of the PCIP. A secondary paper (or stock card) based system was used during power or internet outages. The higher login rates into PCIP in Phase 2 notwithstanding, we felt that utilization may have been higher yet, had web server connectivity been better. As a result of our findings, the medical center recognized the need and subsequently upgraded their internet infrastructure.

As in any environment, we encountered a natural resistance to change. The hospital staff were hesitant to learn a computer-based program, at least initially. We were also cognizant that Haiti has a literacy rate of 40% and a general lack of computer skills. Indeed, the educational background and computer skills of many of the hospital employees were quite variable. Most staff initially had difficulty performing Windows-based computer operations. While training the pharmacy staff to add new medications to the database, we noticed many double entries, spelling errors, and mistyped drug expiration dates. There was a generalized underappreciation regarding the importance of considering such details. The need for correct spelling and dosage entry was important and had to be reiterated during the first several months.

We were able to successfully overcome many of the challenges we encountered, in part because this endeavor took place in the context of continued Mayo Clinic team visits to St. Luke Hospital to assist with patient care and teach Haitian staff. Development of a trusting collaborative relationship motivated the Haitian staff to take ownership and responsibility of the system, and its continued improvement. Secondly, our Mayo Clinic staff were physically present during the initial ‘hands-on’ implementation and teaching of PCIP, during the rapid PDSA cycling system improvement process, as well as during subsequent trips to ‘reinforce’ the education. Reliance on video, remote, or written instructions may not have yielded similar results (7).

The results of our findings are consistent with those of Berger et al. in regards to the need for an uninterrupted supply of drugs while being able to identify, trace, and monitor usage in a third world country (8). Our findings suggest that a simple to use, yet multi-language capable, web-based program can be a successful approach with an off-line alternative to account for the web server inconsistencies that typically occur in developing countries.

After completing a thorough literature search encompassing possible inventory programs currently being used in third world countries we came across only one relevant article. We composed this manuscript as a resource to health care professional providing care in under-resourced communities. We believe this paper provides insight and solutions for quality improvement initiatives within a struggling medical institution and that further control the institution’s medication utilization and pharmaceutical budget.

At 1-year post-implementation, shortages were reported to be minimal, stock levels were at appropriate levels, and drugs could be requested from third parties in a more-timely manner, resulting in a more consistent and cost-effective supply chain of medications. Although more data from the PCIP implementation was not obtained, verbal reports from St. Luke Hospital staff stated the PCIP continued to minimize medication shortages and helped to navigate future usage needs after our research had already been completed. This system may be adapted to other applications such as non-drug supply inventory management. In addition, the system may be amenable to further system improvements by incorporation of bar coding or radio-frequency identification functionality in the future.

## Conclusions

Supply chain logistics, although critical in an emergency, cannot be designed during a disaster or state of emergency (9). We demonstrated that after disaster containment, it was possible and feasible to develop, implement, and sustain a web-based, easy to use PCIP for improved medication supply chain management. The PCIP provides a means for our Haitians colleagues at St. Luke Hospital to manage their medication inventory through a system that allows real-time knowledge of inventory status as well as forecasting, increases availability of medications at the point of care, reduces waste and shortages, and provides a deterrent to drug diversion. The Haitians autonomy and control over their inventory proved to be as important as development of the program itself (10).
